# *Francisella tularensis*: Biology, Pathogenicity, Epidemiology, and Biodefense

**DOI:** 10.3201/eid1312.071169

**Published:** 2007-12

**Authors:** Lars Eisen

**Affiliations:** * Colorado State University, Fort Collins, Colorado, USA

**Keywords:** Tularemia, vector-borne, biodefense, book review

I am pleased to recommend *Francisella tularensis*: Biology, Pathogenicity, Epidemiology, and Biodefense, published by Blackwell Publishing Limited on behalf of the New York Academy of Sciences. This book is a much-needed comprehensive overview of recent research on the causative agent of tularemia, a potentially serious illness that occurs naturally in the United States. *F. tularensis* is a marvel among vector-borne agents of infectious disease. It has a wide geographic distribution (covering most of the Northern Hemisphere) and can be transmitted through a variety of routes including 1) tick or insect bites; 2) handling of infected animals; 3) contact with or ingestion of water, food, or soil; and 4) inhalation of contaminated aerosols. Indeed, *F. tularensis* is notorious for infecting laboratory workers and is a potential bioterrorism agent. The bacterium includes 4 biovars, with the pathogenic type A recently shown to consist of at least 2 subtypes in North America. Natural transmission cycles of *F. tularensis* are complex and poorly understood.

Research on a broad variety of topics was carried out between the 1914 recognition of *F. tularensis* as a disease agent in humans and the 1970s, but few studies focused on this pathogen during the 1980s and 1990s. The recent designations of *F.* *tularensis* by the National Institute of Allergy and Infectious Diseases as a priority A pathogen and a potential bioterrorism agent has resulted in an explosion of new studies on this intriguing pathogen. Primary focal points of these studies have included vaccine development, improved pathogen detection methods, evaluation of the genetic variability of *F. tularensis* biovars commonly associated with human disease, description of the *F. tularensis* genome, and determination of virulence factors. The wealth of information gained from recent studies is elegantly outlined by an impressive group of world leaders in the field of tularemia research. Chapter topics vary from molecular epidemiology, evolution, and ecology of *Francisella* to genetics, genomics, and proteomics of *F. tularensis,* molecular and genetic basis of pathogenesis of *F. tularensis*, animal models, immunity and immunopathogenesis, diagnosis and therapy, vaccine development, and biosafety issues.

Reflecting a disturbing paucity of epidemiologic and field-oriented studies in the past 20 years, especially in North America, only a few chapters include some information on epidemiology, natural transmission cycles of *F. tularensis*, and the role of different transmission routes to humans. As the field of *F. tularensis* and tularemia research moves forward in the 21st century, the explosion of knowledge related to genetics, immunology, and pathogenesis of *F. tularensis* needs to be complemented by renewed studies on natural transmission cycles, transmission routes to humans, and epidemiology.

